# Why Am I Willing to Speak Up? The Impact of Spiritual Leadership on Employee Voice Behavior

**DOI:** 10.3389/fpsyg.2019.02718

**Published:** 2019-12-05

**Authors:** Xuhua Yang, Yuchen Meng, Yong Qiu, Yaqian Feng

**Affiliations:** ^1^School of Labor Economics, Capital University of Economics and Business, Beijing, China; ^2^Business School, Beijing Technology and Business University, Beijing, China; ^3^China International Engineering Consulting Corporation, Beijing, China

**Keywords:** spiritual leadership, employee voice behavior, career success expectation, felt obligation, China

## Abstract

The sustainability of organizations highlights the significance of inspiring employees, especially their inner lives or spiritual identities, and leaders play a vital role. Consistent with social cognitive theory, the purpose of this paper is to explore the linking mechanisms and conditional processes underlying the relationship between spiritual leadership and employee voice behavior. Three-wave survey data were collected from 366 full-time employees and their line managers, and a moderated mediation analysis was performed. The results show that career success expectation fully mediates the relationship between spiritual leadership and employee voice behavior. Additionally, felt obligation is found to indirectly strengthen the effect of spiritual leadership on employee voice behavior via career success expectation. In light of the results, practical implications are provided for managers and future researchers to enhance the sustainability of organizations.

## Introduction

Considerable attention has been devoted to employee voice behavior, which refers to work-related attitudes and behavioral responses that involve speaking up about problems and weaknesses at work and is linked to the sustainability of organizations (Mowbray et al., 2015). At the workplace, it is easier for followers to focus on their work if their leaders listen to their suggestions and concerns (Gupta et al., 2018). However, voice behavior is somewhat risky for employees; thus, employees often hesitate to raise their voice to avoid challenging and upsetting interpersonal relationships, especially with their line managers who typically control resources and rewards (Lepine and Van Dyne, 1998; Gupta et al., 2018). This phenomenon is particularly true among Chinese employees, who often emphasize harmony and worry about offending others (Chen W. et al., 2013). Therefore, some scholars have begun to study voice behavior in the context of China (Yan and Xiao, 2016; Qian et al., 2018; Hu et al., 2018). However, the sample is a main limitation of present studies investigating Chinese employee voice behavior. Yan and Xiao (2016) conducted research in governmental departments, Qian et al. (2018) only chose a logistics company located in northern China in their study and Hu et al. (2018) investigated one state-owned telecommunications company in China. The current study addressed this research gap by collecting data from four Chinese enterprises that are the most representative companies in different industries with different properties. Employee voice behavior has been studied as a key subject of organizational citizenship behaviors (Kwon et al., 2016), and predicting voice behavior has unsurprisingly been difficult; therefore, the potential antecedents of voice behavior are important to determine.

Some research has addressed this topic and suggested several predictors of voice behavior from different perspectives (Lepine and Van Dyne, 1998). Among these predictors, leadership has been considered to play an important role in motivating employees to voice. Many studies have discussed the positive impact of leadership, such as transformational leadership (Liu et al., 2010), paternalistic leadership (Zhang et al., 2015), ethical leadership (Chen and Hou, 2016; Hu et al., 2018), servant leadership (Yan and Xiao, 2016) and empowering leadership (Qian et al., 2018), on employee voice behavior at the workplace. However, the effect of spiritual leadership on employee voice behavior is noticeably absent from this research (Meng et al., 2018), even though prior researchers have observed that altruistic or moral factors guided by spirituality might engender voice behavior (Morrison, 2011), and calling, which is a core factor of spiritual leadership, has also been found to benefit employee voice behavior (Park et al., 2018).

According to Gkinopoulos and Hegarty (2018), the effect of leadership and the behaviors of followers should be considered in a specific historical context. Since 2008, the severe financial crisis that began in the United States widely spread worldwide. Scholars have expressed concern regarding the crisis by noting that the economic crisis was an important stressor and that collective panic could have a potentially negative impact on mental health (Giorgi et al., 2015; Mucci et al., 2016). In this context, a type of leadership concerned with employees’ mental health and spiritual needs was needed to reframe the vision and value and help employees overcome the crisis. Compared to other types of leadership, spiritual leadership pays more attention to the spirit of employees not in a religious sense but in a human-centered view based on self-awareness, life goals, and community engagement (Fernandes Bella et al., 2018). Thus, spiritual leadership is more suitable than other types of leadership for addressing the diverse balance needs of employees and boost morale to improve their motivation and satisfaction (Fry, 2003), which are highly related to post-crisis recovery and the sustainability of organizations. In addition, during the economic crisis, the Chinese government responded to the slowdown of the economy swiftly and forcefully (Yu, 2008), and China’s rapid post-crisis reconstruction showed the world a strong pace of development; thus, it is interesting and meaningful to study the Chinese economic context. In summary, the present study contributes to the voice literature by focusing on spiritual leadership in China. In addition, if spiritual leadership is positively related to voice, we propose further steps to test an explanatory mechanism potentially underlying this relationship.

According to social cognitive theory, factors, such as economic conditions, socioeconomic status, educational level and family background, do not directly affect human behavior. Instead, these factors affect behavior by directly influencing individuals’ desires, self-efficacy beliefs, personal standards, emotional states, and other self-management factors (Bandura, 1986). Therefore, the present study investigates the internal factors explaining the relationship between spiritual leadership and employee voice behavior. Another gap in the spiritual leadership literature is the unclear boundary conditions that strengthen or weaken the effects of spiritual leadership on work outcomes. According to prior research, individual variation can also make a difference in voice (Premeaux and Bedeian, 2003; Crant et al., 2011). Thus, this study considers the moderating effect of individual variation.

### Theoretical Background and Study Hypotheses

#### Spirituality and Leadership

The concept of spiritual leadership is derived from the study of spirituality in the workplace and refers to employees living their values more fully at the workplace and organizations paying more attention to supporting employees’ spiritual growth (Phuong et al., 2018). Gradually, the link between leadership and spirituality has been deeply explored as leaders play an important role in the workplace. Ayranci and Semercioz (2011) noted three ways in which spirituality and leadership are connected. The first category uses spirituality as a tool to achieve organizational goals. The second category posits that spirituality and leadership are independent entities. The third category defines the concept of spiritual leadership. In contrast to the first and second categories, the third category regards spirituality as a leadership trait.

#### Relationship Between Spiritual Leadership and Employee Voice Behavior

Since Hirschman (1970) first proposed the concept of voice in his exit, voice, loyalty (EVL) model and defined voice as “any attempt at all to change, rather than to escape from, an objectionable state of affairs,” the definition has evolved to the individual level, referring to a method of communication between employees and leaders (Lavelle et al., 2010).

Given the risk of voice behaviors, employees’ willingness to engage in such behaviors may largely depend on whether the surrounding environment favors speaking up (Morrison, 2011; Ruck et al., 2017). Accordingly, research has focused on the antecedent factors motivating employees to speak up (Ashford et al., 1998), and researchers have emphasized the importance of contextual factors, especially the role of leaders, in shaping whether employees’ beliefs are safe to voice (Zhu and Akhtar, 2017). Social cognitive theory (Bandura, 1977, 1986) indicates that the interaction among individuals’ cognition, individuals’ behavior, and the environment is constant. In the workplace, leaders’ behavior plays a pivotal role in influencing employees’ behavior (Morrison, 2014); similarly, employees’ voice behavior is gradually formed through social learning and cognition processes in a context in which leaders have a strong demonstration effect on employees (Deng, 2016). Among the positive leadership styles, spiritual leadership emphasizes exerting a subtle influence on employees through the leaders’ values and daily behaviors (Reave, 2005). According to the definition provided by Fry (2003), spiritual leadership comprises values, attitudes, and behavior that can facilitate a sense of spiritual survival among employees. The dimensions of spiritual leadership include vision, hope/faith and altruistic love (Fry, 2003). A clear, stimulating vision drives employees toward the same goals and increases their sense of responsibility to the organization, which, in turn, generates more positive behavior. The hope/faith given by leaders helps employees increase their motivation to achieve their vision while building confidence in the organization and performing actions that are conducive to organizational development. Leaders create an atmosphere of altruistic love at the workplace, which could reduce the uneasiness of employees, enhance their sense of belonging, and help them express their true ideas. Altogether, spiritual leadership often utilizes spirituality and influence to create a vision and establish an organizational culture in which organization members experience a sense of calling and membership (Fry, 2003) and then engage in more organizational citizenship behaviors, including voice behavior (Hunsaker, 2016). In addition, spiritual leadership is beneficial for increasing the organizational identity of employees and improving the relationship between leaders and employees, which creates a better atmosphere in which individuals can speak their opinion (Meng et al., 2018; Qiu et al., 2019). Thus, we propose the following hypothesis:

Hypothesis 1: Spiritual leadership is positively related to employee voice behavior.

#### Mediating Effect of Career Success Expectation on the Spiritual Leadership-Employee Voice Behavior Relationship

Based on social cognitive theory, external factors often influence human behavior by affecting people’s internal factors (Bandura, 1986). Therefore, this study speculated that there is an internal factor that mediates the relationship between spiritual leadership and employee voice behavior. In previous studies, some psychological variables, such as calling, meaning, self-esteem and self-efficacy, are found to mediate the relationship between spiritual leadership and organizational outcomes (Chen et al., 2012; Javanmard, 2012). These findings illustrate the mediating role of employees’ self-awareness in the relationship between leadership and subordinates’ behaviors. However, as an important internal factor, individuals’ expectations lack attention. According to educational and psychological scholars, when expectations of success are high, individuals are more likely to persist in work, gain satisfaction and make some achievements (Feather, 1961; Nurmi et al., 2003). At the workplace, the expectation of career success often reflects external results, such as promotions and career choices (Seibert et al., 2001). Therefore, we propose that career success expectation, as a type of aspiration, represents a connection between spiritual leadership and employee voice behavior. Career success expectation is defined as expected future work achievements (Lin et al., 2012). For individuals, positive career success expectations lead to better career management (Stephens et al., 1998; Rousseau, 2001), and employees may engage in more beneficial behaviors at work.

Furthermore, social cognitive theory posits that career success expectations include the expected results and the value of these results to the individual, both of which may be affected by significant others (e.g., leaders). Lent et al. (1994) established a social cognitive model of career development to highlight the importance of learning and developing psychological relationships for fostering optimistic career success expectations. Spiritual leadership delivers an inspiring vision to followers to motivate them to achieve their own expectations of success, which may increase their intrinsic motivation and career success expectations. Hence, spiritual leadership may be associated with voice via the pathway involving career success expectation. Thus, we hypothesize the following:

Hypothesis 2: Career success expectation mediates the relationship between spiritual leadership and employee voice behavior.

#### Moderation of Felt Obligation

When individuals have different perceptions and traits, they may behave differently. Thus, although career success expectation may affect the behaviors of employees, such behaviors also differ due to individual psychological differences (Premeaux and Bedeian, 2003; Crant et al., 2011). Spiritual leadership delivers a clear vision to employees and motivates employees to achieve this vision with positive faith; furthermore, this type of leadership could create a mutually supportive, altruistic atmosphere to meet employees’ spiritual needs at the workplace. As a result, when employees gain spiritual and emotional resources in the organization, they may generate a sense of responsibility for the organization (Cropanzano and Mitchell, 2005), which can effectively link the employees’ existence value with the development of the organization such that employees have higher expectations and work motivation. Thus, we propose that felt obligation plays a moderator role in the proposed model.

Felt obligation is an important self-concept reflecting the idea that individuals can sense responsibility and obligation for their work results. This concept is similar to the higher approach of a reciprocal psychological contract, which leads to differences among employees (Fuller et al., 2010). Prior studies have indicated that certain individuals with greater felt obligation often exert themselves by working long, assiduous hours to meet job demands and achieve success, with positive implications for their jobs. Specifically, employees with high felt obligation can effectively connect their self-value with organizational needs and regard themselves as an important part of the organization; thus, such employees are willing to take actions that are beneficial to the organization, their colleagues, and customers (Eisenberger et al., 2001). In contrast, when felt obligation is at a low level, employees may engage in less extra-role behavior (Liang, 2014). Furthermore, felt obligation has been widely tested as an effective predictor of voice behavior (Morrison, 2014; Zhu and Akhtar, 2017). Therefore, we hypothesize that employees with high felt obligation are more proactive and achieve their career success expectation by committing to the mission and strategic objectives of the organization, contributing their wisdom and paying more attention to their work, which may result in voice behavior to improve their development and organization.

Hypothesis 3: Felt obligation positively moderates the relationship between career success expectation and employee voice behavior such that this relationship is more significant when felt obligation is high than when it is low.

Assuming that felt obligation moderates the association between career success expectation and voice, employees’ felt obligation is also likely to conditionally affect the strength of the indirect relationship between spiritual leadership and employee voice behavior. Because we predict a strong relationship between career success expectation and voice when felt obligation is high, we expect the following:

Hypothesis 4: Felt obligation positively moderates the indirect effect of spiritual leadership on employee voice behavior via career success expectation such that the indirect effect is stronger when felt obligation is high than when it is low.

**FIGURE 1:**
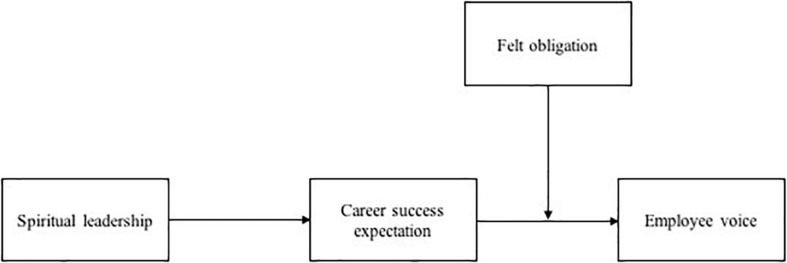
Hypothesized model.

Thus, we propose a moderated-mediation model, and the relationships among spiritual leadership, employee voice behavior, career success expectation and felt obligation are shown in Figure 1.

## Materials and Methods

### Procedures and Participants

The study was performed using a questionnaire survey. The eligible participants were full-time employees and their line managers from four Chinese companies. To ensure the diversity and representativeness of the samples, two selected companies are state-owned corporations, and the other companies are private companies; in addition, these companies are the most representative companies in different industries. Additionally, all procedures performed in studies involving human participants were performed in accordance with the ethical standards of the institutional and national research committee and the 1964 Helsinki Declaration and its later amendments or comparable ethical standards, and written informed consent was obtained from all subjects. The data were collected from the target participants at three time points at 2 months intervals to reduce the effects of common method bias (Podsakoff et al., 2012).

The first survey (T1) was administered to the employees to investigate their perspectives regarding spiritual leadership and empowering leadership. We also collected demographic information at this time. One month later, the second survey (T2) collected information regarding the employees’ career success expectations, felt obligation, psychological safety and guanxi. During the final wave (T3), the managers evaluated the followers’ voice behavior. We contacted the target employees’ line managers to assess these employees’ voice behavior. We received 452 responses in T1, but 411 responses remained after we eliminated 41 responses due to low-effort responding (i.e., above 80% of the same answers or quick answers). Then, 379 employees completed the questionnaires in T2. Finally, 366 matched responses available from T3 were used for the analyses. Of this final sample, the respondents were primarily (*n* = 263, 71.9%) men and aged 38.14 years (*SD* = 9.60) on average. The analyses were computed using the Statistical Package for Social Sciences version 24.0 (IBM SPSS Statistics 24, SPSS Inc., Chicago, IL, United States) and the AMOS statistical package version 22.0 (Arbuckle, 2010).

### Measures

The original questionnaires measuring all variables were in English. In accordance with Brislin’s back-translation procedure (Brislin, 1970), the original survey items were translated into Chinese and then back-translated into English.

#### Spiritual Leadership

The construct of spiritual leadership was measured by 17 items extracted from the 26-item Spiritual Leadership Scale developed by Fry et al. (2005). The Cronbach’s alpha of this scale was 0.97. Sample items included in this scale are as follows: “I understand and am committed to my organization’s vision (vision)”; “I have faith in my organization and I am willing to ‘do whatever it takes’ to ensure that it accomplishes its mission (hope/faith)”; and “My organization really cares about its people (altruistic love).”

#### Employee Voice Behavior

We measured employee voice behavior with 3 items adapted from Madrid et al. (2015). To ensure the objectivity of the data, we changed the items from self-rated to leader-rated. The Cronbach’s alpha of this scale was 0.92. A sample item is “This employee made recommendations concerning issues that affect his work.”

#### Career Success Expectation

The career success expectation measure was a 3-item scale developed by Seibert et al. (2001) and modified by Lin et al. (2012) to adapt to the Chinese culture. The Cronbach’s alpha of this scale was 0.94. A sample item is “I expect the likelihood of my promotion is high if I work in the firm.”

#### Felt Obligation

We measured felt obligation using a 7-item scale developed by Eisenberger et al. (2001), which assessed employees’ felt obligation for organizational development. The Cronbach’s alpha of this scale was 0.87. A sample item is “I feel a personal obligation to do whatever I can to help my company achieve its goals.”

#### Control Variables

Consistent with previous research concerning voice at work (Morrison, 2011), we incorporated the following demographic variables as control variables into our model: the participants’ gender, age, and organizational tenure.

In addition, as described by Atinc et al. (2012) and Becker (2005), appropriate control variables should be included in organizational research to rule out the possibility of endogeneity (Antonakis et al., 2010). Moreover, recent leadership research (Martinko et al., 2018) has identified a substantial degree of conceptual and empirical overlap between the constructs of positive leaderships. Furthermore, a recent study investigating the use of control variables in leadership research (Bernerth et al., 2018) highlights the importance of including requisite control variables in studies of leadership. Thus, we measured other leadership types simultaneously in this study along with all measured leadership styles and found that empowering leadership and spiritual leadership are the most relevant (*r* = 0.520, *p* < 0.000); thus, we chose empowering leadership as a control variable. Theoretically, empowering leadership and spiritual leadership have partial similarities (Russell, 2001). From the theoretical basis perspective, empowering leadership developed from social cognitive theory (Pearce and Sims, 2002), while social cognitive theory is an important theoretical basis of spiritual leadership (Fry, 2003). This theory posits that leaders can lead their followers to learn positive behaviors by leading by example, which also endowed employees more initiative. Regarding its characteristics, empowerment is an important means by which spiritual leadership works (Fry, 2003), while empowering leadership has also been found to have some spiritual characteristics (Keyes et al., 1999; Hermans and Koerts, 2013). Regarding the positive effects, some studies have confirmed that empowering leadership positively predicts employee voice behavior (Qian et al., 2018); therefore, it is necessary to consider the potential impact of empowering leadership. We measured empowering leadership using the scale developed by Ahearne et al. (2005).

Similar suspicions exist regarding the role of career success expectations because many other mechanisms related to internal states have been confirmed. The link between leadership and employee voice behavior has been studied from the perspective of cost–benefit analyses and the self-concept (Duan et al., 2017). The cost-benefit analysis perspective highlights that leaders could cultivate psychological safety among employees such that they are willing to engage in risk-taking behaviors (Liang et al., 2012). In addition, the self-concept perspective indicates that leaders could build a strong relational self that motivates employees to voice (Liu et al., 2010). Thus, in this study, we chose psychological safety and guanxi as control variables to exclude the possible effects of employees’ psychological factors and the relationship between leaders and followers. Psychological safety is an important variable explaining voice behavior (Liang et al., 2012). Because voice behavior may cause high personal costs for employees, leading them to fear speaking out (Li et al., 2014), scholars generally believe that the key to promoting voice is to guide employees to perceive safety. Additionally, existing research has found that psychological safety is an important mediator variable predicting voice behavior (Walumbwa and Schaubroeck, 2009; Liang et al., 2012; Cheng et al., 2013; Hu et al., 2018); thus, we included psychological safety as a control variable. In addition to psychological factors, the relationship between leaders and employees is a crucial factor predicting voice behavior (Van Dyne et al., 2008). Scholars have used leader member exchange to describe this relationship, however, because this research focuses on the Chinese context, we prefer to use “guanxi.” Guanxi is defined as personal connections bound by implicit psychological contracts to exchange reciprocity, nurture mutual commitment, and aim for long-term relationships (Chen C.C. et al., 2013). Guanxi is distinct because Chinese leaders and followers do not clearly distinguish between workplace and after-work relationships and often mix the two areas when building relationships (Wang et al., 2019). According to Wang et al. (2019), exchange reciprocity may prompt followers in close guanxi with leaders to speak up; furthermore, compared with those who have poor guanxi with their superiors, employees with close guanxi with their leaders encounter less risk from voice. Additionally, guanxi has been proven to play a mediating role in the studies investigating the antecedents of voice behavior (Song et al., 2017; Yan, 2018). In summary, “psychological safety” and “guanxi” were controlled for in this study to rule out the possibility of endogeneity. We measured psychological safety using the scale developed by Liang et al. (2012) and guanxi using the scale developed by Chen et al. (2009).

## Results

### Preliminary Analyses

We conducted a confirmatory factor analysis (CFA) using AMOS 22 to test the validity. To assess the model fit, the significance of the chi-square is regarded as the criterion; however, the chi-square is simply affected by the sample size. We also examined other fit indexes [i.e., comparative fit index (CFI), incremental fit index (IFI), root mean square error of approximation (RMSEA), and root mean square residual (RMR)]. Table 1 shows the results and comparisons of the confirmatory factor analyses; it can be observed that the chi-square of the other models (M2–M5) significantly increases in contrast to that of the four-factor model (M1) and that the four-factor model (M1) is apparently better in the other fit indices; thus, it can be concluded that these four variables empirically differ. Moreover, M1 shows a satisfactory absolute fit as follows: χ^2^ = 1156.974; χ^2^/df = 2.922, CFI = 0.934; IFI = 0.934; RMSEA = 0.073; and RMR = 0.034. The results of the CFA show that the proposed model fit the data as the normalized chi-square (chi-square/degrees of freedom) of the CFA model was smaller than the recommended value of 3.0, the CFI and IFI were greater than 0.90, the RMSEA was smaller than 0.08, and the RMR was smaller than 0.05.

**TABLE 1:** Confirmatory factor analyses.

**Model**	**χ^2^**	***df***	**χ^2^/*df***	**CFI**	**IFI**	**RMSEA**	**RMR**
M1	1156.974	396	2.922	0.934	0.934	0.073	0.034
M2	2092.417	399	5.244	0.853	0.853	0.108	0.062
M3	2684.094	401	6.694	0.802	0.803	0.125	0.064
M4	3087.047	401	7.698	0.767	0.768	0.135	0.166
M5	4468.892	405	11.034	0.648	0.649	0.166	0.116

**n* = *366. M1: Spiritual leadership; career success expectation; felt obligation; employee voice behavior. M2: Spiritual leadership; career success expectation* + *felt obligation; employee voice behavior. M3: Spiritual leadership; career success expectation* + *felt obligation* + *employee voice behavior. M4: Spiritual leadership* + *career success expectation* + *felt obligation; employee voice behavior. M5: Spiritual leadership* + *career success expectation* + *felt obligation* + *employee voice behavior.**

The convergent validity of the model can be validated by the criterion that all average variances extracted (AVEs) exceed 0.50 (Fornell and Larcker, 1981). Furthermore, a component reliability (CR) of at least 0.70 can indicate convergent validity. According to the analytical results of AVE and CR, all AVEs of the research variables were larger than 0.50 as follows: 0.76 (spiritual leadership), 0.79 (employee voice behavior), 0.85 (career success expectation), and 0.58 (felt obligation). Additionally, all CRs exceeded 0.90 as follows: 0.98 (spiritual leadership), 0.92 (employee voice behavior), 0.94 (career success expectation), and 0.90 (felt obligation). These results reveal that the convergent validity of the research variables was satisfactory.

### Descriptive Analyses

Table 2 presents the means, standard deviations, and correlations of the studied variables. Consistent with our hypotheses, spiritual leadership was positively correlated with employee voice behavior (*r* = 0.376, *p* < 0.01) and career success expectation (*r* = 0.305, *p* < 0.01), which was positively correlated with employee voice behavior (*r* = 0.525, *p* < 0.01). Felt obligation was significantly and positively related to employee voice behavior (*r* = 0.433, *p* < 0.01).

**TABLE 2:** Means, standard deviations, and correlations.

**Variables**	**Mean**	***SD***	**1**	**2**	**3**	**4**	**5**	**6**	**7**	**8**	**9**
1. Gender^a^	0.28	0.45									
2. Age	38.14	9.60	–0.036								
3. Organizational tenure	7.71	7.65	–0.014	0.397^∗∗^							
4. Empowering leadership	3.93	0.81	–0.076	–0.038	–0.080						
5. Psychological safety	4.04	0.81	–0.032	–0.041	–0.080	0.454^∗∗^					
6. Guanxi	3.78	0.73	–0.050	–0.173^∗∗^	–0.058	0.077	0.012				
7. Spiritual leadership	4.09	0.74	–0.030	–0.043	–0.161^∗∗^	0.520^∗∗^	0.449^∗∗^	0.052			
8. Career success expectation	3.34	0.90	–0.066	–0.091	–0.147^∗∗^	0.192^∗∗^	0.340^∗∗^	–0.030	0.305^∗∗^		
9. Felt obligation	3.96	0.72	0.035	0.026	−0.123^∗^	0.330^∗∗^	0.419^∗∗^	0.006	0.451^∗∗^	0.373^∗∗^	
10. Employee voice behavior	3.78	0.72	–0.077	–0.085	–0.097	0.294^∗∗^	0.545^∗∗^	0.037	0.376^∗∗^	0.525^∗∗^	0.433^∗∗^

**n* = *366. ^*a*^0* = *male, 1* = *female. ^∗^p* < *0.05. ^∗∗^p* < *0.01.**

### Hypothesis Testing

We tested the hypotheses in two interlinked steps. Tables 3, 4 present the mediation effects of career success expectation. First, we tested the direct effect of spiritual leadership on employee voice behavior and the mediation role of career success expectations. As shown in Table 3, the results reveal that when gender, age, organizational tenure, empowering leadership, psychological safety and guanxi were held constant, spiritual leadership (*B* = 0.228, *p* < 0.01) served as a significant direct predictor of career success expectation. After controlling for the effects of spiritual leadership, career success expectation also had a significant effect on employee voice behavior (*B* = 0.293, *p* < 0.001). Furthermore, as shown in Table 4, by using the bootstrapping method for further calculation, we found a significant indirect effect (95% CI = [0.023, 0.122]) and an insignificant direct effect (95% CI = [-0.002, 0.190]). Thus, career success expectation completely mediates the relationship between spiritual leadership and employee voice behavior. These results support H1-2 (see Tables 3,4).

**TABLE 3:** Mediation effects of career success expectation.

**Variable**	**career success**	**employee voice**	
	**expectation as a**	**behavior as a**	
	**dependent variable**	**dependent variable**	
	**Model 1**	**Model 2**	**Model 3**
Constant	1.905^∗∗∗^	1.614^∗∗∗^	1.056^∗∗∗^
Gender	–0.144	–0.102	–0.060
Age	–0.005	–0.004	–0.003
Organizational tenure	–0.010	–0.001	0.002
Empowering leadership	–0.028	–0.010	–0.002
Psychological safety	0.288^∗∗∗^	0.421^∗∗∗^	0.336^∗∗∗^
Guanxi	–0.069	0.010	0.031
Spiritual leadership	0.228^∗∗^	0.161^∗∗^	0.094
Career success expectation			0.293^∗∗∗^
F	10.029^∗∗∗^	24.495^∗∗∗^	34.285^∗∗∗^
*R*^2^	0.165	0.325	0.436

**n* = *366.*^∗∗^*p* < 0.01. ^∗∗∗^*p* < 0.001.*

**TABLE 4:** Mediation effects of career success expectation.

	**Direct and indirect effects of spiritual leadership on employee voice behavior**			
	
	**Effect**	**Boot SE**	**Boot LLCI**	**Boot ULCI**
Direct effect	0.094	0.049	–0.002	0.190
Indirect effect	0.067	0.025	0.023	0.122

**n* = *366.**

To support the simple moderation hypothesis (H3), the coefficients of the interaction term in the mediator model should be significant. Table 5 shows the moderating effect of felt obligation, and the results show that after controlling for the effects of the demographic variables, empowering leadership, psychological safety and guanxi, there was an interaction effect between career success expectation and felt obligation on employee voice behavior (*B* = 0.11, *SE* = 0.04, *t* = 2.982, *p* < 0.01), supporting H3.

**TABLE 5:** Moderation and moderated mediation effects of felt obligation.

**Mediator variable model with employee voice behavior as the dependent variable**						
**Variable**	***B***	**S.E.**	***t***	***P***	**LLCI**	**ULCI**
Constant	2.576	0.281	9.168	0.000	2.023	3.129
Career success expectation	0.262	0.035	7.413	0.000	0.192	0.331
Felt obligation	0.197	0.048	4.148	0.000	0.104	0.291
CSE × FO	0.113	0.038	2.982	0.003	0.039	0.188
Gender	–0.075	0.064	–1.178	0.240	–0.201	0.050
Age	–0.004	0.003	–1.259	0.209	–0.011	0.002
Organizational tenure	0.002	0.004	0.440	0.660	–0.006	0.010
Empowering leadership	0.005	0.040	0.136	0.892	–0.073	0.084
Psychological safety	0.297	0.043	6.915	0.000	0.212	0.381
Guanxi	0.032	0.040	0.799	0.425	–0.046	0.110
*R*	0.680					
*R*^2^	0.462					

**n* = *366.**

**FIGURE 2:**
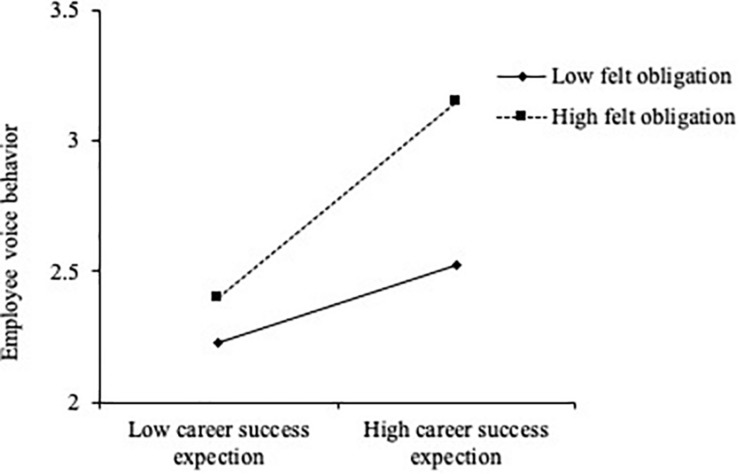
Interaction effect between career success expectation and felt obligation on employee voice behavior. Low felt obligation is defined as at least one standard deviation below the mean; high felt obligation is defined as at least one standard deviation above the mean.

To fully support H3, we applied conventional procedures to plot the simple slopes at one standard deviation above and below the mean of felt obligation. Figure 2 presents the interaction effect between career success expectation and felt obligation on employee voice behavior; as shown in the figure, when felt obligation was higher, the relationship between career success expectation and employee voice behavior was stronger.

Table 6 presents the results of the moderated-mediation effects of felt obligation. As anticipated, the indirect effect (CI = [0.001, 0.070]) was significant. Furthermore, the results show that the indirect effect of spiritual leadership on employee voice behavior through career success expectation significantly varied across the high and low levels of felt obligation, supporting H4. Thus, we conclude that the moderated mediation was supported.

**TABLE 6:** Moderation and moderated mediation effects of felt obligation.

**Value of felt obligation**	**Indirect effect**	**Boot SE**	**Boot LLCI**	**Boot ULCI**
**Conditional indirect effect as a function of felt obligation**				
MEAN-1 SD (-0.72)	0.040	0.020	0.010	0.091
MEAN(0)	0.059	0.023	0.020	0.112
MEAN + 1 SD (0.72)	0.078	0.031	0.026	0.153

**Mediator**	**Index**	**Boot SE**	**Boot LLCI**	**Boot ULCI**

**Index of Moderated Mediation**				
Career success expectation	0.026	0.017	0.001	0.070

**n* = *366.**

## Discussion

### Main Findings

The purpose of this paper is to explore the linking mechanisms and conditional processes underlying the relationship between spiritual leadership and employee voice behavior. The results revealed three main points. First, spiritual leadership played a significant role in predicting career success expectation and employee voice behavior. Second, career success expectation played a fully mediating role in the process by which spiritual leadership affects employee voice behavior. Finally, felt obligation played a moderating role in this model; specifically, the indirect effect of spiritual leadership on employee voice behavior through career success expectation was stronger among employees with a higher level of felt obligation. These findings have implications for research concerning spiritual leadership and employee voice behavior.

### Theoretical Implications

This study contributed to the literature concerning spiritual leadership and employee voice behavior based on social cognitive theory. With societal and economic development, work time occupies most of our life; in turn, our demands have increased, and we aim to find spiritual survival in the workplace (Fairholm, 1996; Fry, 2003). In this context, traditional bureaucratic leadership may find it difficult to satisfy individuals’ needs, and the role of spiritual leadership must be taken seriously. Nevertheless, research investigating spiritual leadership in the Chinese context is deficient. The theoretical model proposed in this study enriches empirical research in the fields of spiritual leadership and employee voice behavior.

Although spiritual leaders’ demonstration effect may directly influence followers’ behaviors, more likely, individuals’ attitudes and value are primarily affected by leaders. Thus, this study introduced career success expectation to explain how individuals transform external effects into their behaviors through learning and absorbing. Through an empirical analysis, we found that career success expectation played a mediating role in the process by which spiritual leadership influences employee voice behavior and suggest that the external effect of leaders influences individuals’ aspirations and then shapes their behaviors. This finding clarifies the complicated connection between leaders’ effects and followers’ behaviors. Furthermore, this study enriches research concerning career success expectation. In the field of career development, career success is an important topic, but career success expectation lacks attention (Cho and Ryu, 2016). However, as a positive psychological construct, career success expectation is important for career choices, career transitions, and education investment (Vos et al., 2009). Understanding followers’ career success expectation can enable more effective human resource management measures for individual career success (Stephens et al., 1998).

Finally, this study essentially found that felt obligation moderated the indirect effect of career success expectation in the relationship between spiritual leadership and employee voice behavior. As an important self-concept, felt obligation is formed by individuals’ thoughts and perceptions of themselves and reflects their sense of responsibility for their work. In our study, when experiencing a high level of felt obligation and a strong feeling of belonging to the organization because of spiritual leadership, employees with high career success expectation utilized their energy to cope with job demands and voice their thoughts to develop the organization. Thus, our research provides new knowledge for the field of organizational development by clarifying the boundary conditions through which spiritual leadership and career success expectation can effectively contribute to employee voice behavior.

### Practical Implications

In an era of globalization and information, external turmoil has profoundly affected the development of organizations. In this context, more organizations have redeployed to maintain their corporate sustainability (Ambreen et al., 2019). The present study has implications for enhancing the sustainability of organizations. Because voice behavior has positive effects on seeking out problems and putting forward new ideas at work, it is highly useful for realizing the sustainability of organizations (Mowbray et al., 2015). To motivate voice behavior, organizations should encourage leaders to engage in spiritual leadership by developing their values and behaviors enhancing the demonstration effect. Organizations can also employ the sets of spiritual behaviors measured in the present study as a tool when rewarding managers. Furthermore, we propose specific suggestions. The first suggestion is to be far-sighted. Leaders should exert the function of vision in enterprises, clearly convey this vision to staff, and ensure that employees fully understand the enterprise’s values and vision. These actions make it easier for leaders to be trusted and respected by followers and promote followers to pursue consistent goals. The second suggestion is to be good at motivating. The power of hope and faith is strong in organizations (Fry, 2003), and vision may allow followers to look forward to the future. Hope and faith facilitate positive expectations and increase motivation; therefore, leaders must be deeply concerned about the spiritual needs of employees and meet these needs within reason. The final suggestion is to create a loving atmosphere. Altruistic love is helpful for improving the organizational atmosphere and allows employees to feel understood and appreciated. A harmonious atmosphere leads to good organizational behavior among employees.

In addition, supervisors should help followers form positive career success expectations to lead to better career development. Additionally, insight into employees’ career success expectations could render human resource management more effective. Furthermore, managers must pay more attention to the empowerment and cultivation of felt obligation to promote employee responsibility and realize organizational sustainability.

### Limitations and Future Research

First, although the present study provided new insight into the relationships among spiritual leadership, career success expectation, felt obligation and employee voice behavior, some limitations need to be addressed. Although the variables were measured at different times in a temporal order matching their place in the proposed model, the findings do not ensure strong causal relations among the study variables. Furthermore, the concepts of spiritual leadership and employee voice behavior are not static; thus, longitudinal designs or experiments should be designed to better establish causality in the future.

Second, more efforts are needed to unfold the spiritual leadership–voice association and the influence of the relationship situation. We recommend the introduction and integration of diverse perspectives and theories to explain this phenomenon. Although career success expectation was shown to be an important mediator in the present study, future research is encouraged to investigate other potential intervening variables linking spiritual leadership to employee voice behavior. In addition, the choice of the control variables is another shortcoming in this study, and controlling more relevant variables should be considered to rule out the possibility of endogeneity in future research.

Third, the conclusions refer to a specific economic context and a specific geographical area because of the limited sample. To some extent, our sample from China limits the generalizability of our conclusions. The Chinese culture emphasizes harmony; as a result, Chinese employees find it difficult to voice their real thoughts in the workplace. Hence, the results may differ in other countries because of cultural differences. Thus, we hope that researchers from other cultures can replicate our research and consider contextual and cultural factors when interpreting our results.

## Conclusion

From the perspective of social cognitive theory, the present study examined the relationships among spiritual leadership, employee voice behavior, career success expectation and felt obligation. We found that spiritual leadership has motivational influences on followers by promoting their sense of calling and membership. Furthermore, spiritual leadership delivers an inspiring vision to followers and motivates them to achieve their own expectations of success, which may elevate their intrinsic motivation and career success expectation. Subsequently, employees develop positive attitudes and behaviors toward the organization and, therefore, are willing to engage in voice behavior. Altogether, these findings indicate the strong incentive function of spiritual leadership and show how mental health and rebuilding employees’ confidence were achieved after the economic crisis. Additionally, we studied this issue in a strongly developing economic system, i.e., the Chinese system, which not only enriches relevant research in China but also provides experience for other Eastern countries or developing countries.

## Data Availability Statement

The datasets generated for this study are available on request to the corresponding author.

## Ethics Statement

All procedures performed in studies involving human participants were performed in accordance with the ethical standards of the institutional and/or national research committee and the 1964 Helsinki Declaration and its later amendments or comparable ethical standards with written informed consent from all subjects. This research was approved by the Human Research Ethics Committee at School of Labor Economics, Capital University of Economics, and Business.

## Author Contributions

XY and YQ designed the research and collected the data for the study. YM and YF analyzed the data and drafted the work. All authors critically reviewed and approved the final version of this manuscript.

## Conflict of Interest

YF was employed by company China International Engineering Consulting Corporation. The remaining authors declare that the research was conducted in the absence of any commercial or financial relationships that could be construed as a potential conflict of interest.

## References

[B1] Ahearne M. Mathieu J. Rapp A. (2005). To empower or not to empower your sales force? An empirical examination of the influence of leadership empowerment behavior on customer satisfaction and performance. *J. Appl. Psychol.* 90 945–955. 10.1037/0021-9010.90.5.945 16162066

[B2] Ambreen M. Muhammad N. A. Usman T. Kirk C. (2019). Transformational changes and sustainability: from the perspective of identity, trust, commitment, and withdrawal. *Sustainability* 11:3159 10.3390/su11113159

[B3] Antonakis J. Bendahan S. Jacquart P. Lalive R. (2010). On making causal claims: a review and recommendations. *Leadersh. Quart.* 21 1086–1120. 10.1016/j.leaqua.2010.10.010

[B4] Arbuckle J. L. (2010). *). IBM SPSS Amos 19 User’s Guide.* Crawfordville, FL: Amos Development Corporation.

[B5] Ashford S. J. Rothbard N. P. Piderit S. K. Dutton J. E. (1998). Out on a limb: the role of context and impression management in selling gender-equity issues. *Admin. Sci. Quart.* 43 23–57. 10.2307/2393590

[B6] Atinc G. Simmering M. J. Kroll M. J. (2012). Control variable use and reporting in macro and micro management research. *Organ. Res. Methods* 15 57–74. 10.1177/1094428110397773 29787210

[B7] Ayranci E. Semercioz F. (2011). The relationship between spiritual leadership and issues of spirituality and religiosity: a study of top turkish managers. *Int. J. Bus. Manag.* 6 1833–8119. 10.5539/ijbm.v6n4p136

[B8] Bandura A. (1977). *Social Learning Theory.* Englewood Cliffs, NJ: Prentice-Hall.

[B9] Bandura A. (1986). *Social Foundations of Thought and Action: A Social Cognitive Theory.* Englewood Cliffs, NJ: Prentice-Hall.

[B10] Becker T. E. (2005). Potential problems in the statistical control of variables in organizational research: a qualitative analysis with recommendations. *Organ. Res. Methods* 8 274–289. 10.1177/1094428105278021

[B11] Bernerth J. B. Cole M. S. Taylor E. C. Walker H. J. (2018). Control variables in leadership research: a qualitative and quantitative review. *J. Manag.* 44 131–160. 10.1177/0149206317690586 30578019

[B12] Brislin R. W. (1970). Back-translation for cross-cultural research. *J. Cross Cult. Psychol.* 1 185–216. 10.1177/135910457000100301

[B13] Chen C. Yang C. Li C. (2012). Spiritual leadership, follower mediators, and organizational outcomes: evidence from three industries across two major Chinese societies. *J. Appl. Soc. Psychol.* 42 890–938. 10.1111/j.1559-1816.2011.00834.x

[B14] Chen C. C. Chen X. P. Huang S. (2013). Chinese guanxi: an integrative review and new directions for future research. *Manag. Organ. Rev.* 9 167–207. 10.1111/more.12010

[B15] Chen W. Duan J. Tian X. (2013). Why do not employees voice: a chinese culture perspective. *Adv. Psychol. Sci.* 21 905–913. 10.3724/SP.J.1042.2013.00905

[B16] Chen S. Y. Hou Y. H. (2016). The effects of ethical leadership, voice behavior and climates for innovation on creativity: a moderated mediation examination. *Leadersh. Quart.* 27 1–13. 10.1016/j.leaqua.2015.10.007

[B17] Chen Y. Friedman R. Yu E. Fang W. Lu X. (2009). Supervisor-subordinate guanxi: developing a three-dimensional model and scale. *Manag. Organ. Rev.* 5 375–399. 10.1111/j.1740-8784.2009.00153.x

[B18] Cheng J. W. Lu K. M. Cheung Y. H. Kuo J. H. (2013). Psychological safety serves as mediator between person-group fit and voice behaviour. *Int. J. Manag. Enterp. Dev.* 12 296–309. 10.1504/ijmed.2013.056434

[B19] Cho T. Ryu K. (2016). The impacts of family-work conflict and social comparison standards on Chinese women faculties’ career expectation and success, moderating by self-efficacy. *Career Dev. Int.* 21 299–316. 10.1108/CDI-11-2015-0146

[B20] Crant J. M. Kim T. Y. Wang J. (2011). Dispositional antecedents of demonstration and usefulness of voice behavior. *J. Bus. Psychol.* 26 285–297. 10.1007/s10869-010-9197-y

[B21] Cropanzano R. Mitchell M. S. (2005). Social exchange theory: an interdisciplinary review. *J. Manag.* 31 874–900. 10.1177/0149206305279602

[B22] Deng Z. (2016). The impact of spiritual leadership on employee’s job engagement. *Econ. Manag.* 38 181–189. 10.19616/j.cnki.bmj.2016.04.017

[B23] Duan J. Li C. Xu Y. Wu C. H. (2017). Transformational leadership and employee voice behavior: a pygmalion mechanism. *J. Organ. Behav.* 38 650–670. 10.1002/job.2157

[B24] Eisenberger R. Armeli S. Rexwinkel B. Lynch P. D. Rhoades L. (2001). Reciprocation of perceived organizational support. *J. Appl. Psychol.* 86 42–51. 10.1037/0021-9010.86.1.42 11302232

[B25] Fairholm G. W. (1996). Spiritual leadership: fulfilling whole-self needs at work. *Leadersh. Org. Dev. J.* 17 11–17. 10.1108/01437739610127469

[B26] Feather N. T. (1961). The relationship of persistence at a task to expectation of success and achievement related motives. *J. Abnorm. Soc. Psychol.* 63 552–561. 10.1037/h0045671 13891927

[B27] Fernandes Bella R. Gonçalves Quelhas O. Toledo Ferraz F. Soares Bezerra M. (2018). Workplace spirituality: sustainable work experience from a human factors perspective. *Sustainability* 10 1–13. 10.3390/su1006188730607262

[B28] Fornell C. Larcker D. F. (1981). Evaluating structural equation models with unobservable variables and measurement error. *J. Mark. Res.* 18 39–50. 10.2307/3150980

[B29] Fry L. W. (2003). Toward a theory of spiritual leadership. *Leadersh. Quart.* 14 693–727. 10.1016/j.leaqua.2003.09.001 7798354

[B30] Fry L. W. Vitucci S. Cedillo M. (2005). Spiritual leadership and army transformation: theory, measurement, and establishing a baseline. *Leadersh. Quart.* 16 835–862. 10.1016/j.leaqua.2005.07.012

[B31] Fuller J. B. Marler L. E. Hester K. (2010). Erratum: promoting felt responsibility for constructive change and proactive behavior: exploring aspects of an elaborated model of work design. *J. Organ. Behav.* 27 1089–1120. 10.1002/job.408

[B32] Giorgi G. Arcangeli G. Mucci N. Cupelli V. (2015). Economic stress in the workplace: the impact of fear of the crisis on mental health. *Work* 51 135–142. 10.3233/WOR-141844 24594539

[B33] Gkinopoulos T. Hegarty P. (2018). Commemoration in crisis: a discursive analysis of who ‘we’ and ‘they’ have been or become in ceremonial political speeches before and during the Greek financial downturn. *Br. J. Soc. Psychol.* 57 591–609. 10.1111/bjso.12244 29453781

[B34] Gupta M. Ravindranath S. Kumar Y. (2018). Voicing concerns for greater engagement: does a supervisor’s job insecurity and organizational culture matter? *Evid. Based HRM.* 6 54–65. 10.1108/EBHRM-12-2016-0034

[B35] Hermans C. A. M. Koerts E. (2013). Towards a model of influence of spirituality on leadership: empirical research of school leaders on catholic schools in the Netherlands. *J. Beliefs Val.* 34 204–219. 10.1080/13617672.2013.801685

[B36] Hirschman A. O. (1970). *Exit, Voice, and Loyalty: Responses to Decline in Firms, Organizations, and States.* Cambridge, MA: Harvard University Press.

[B37] Hu Y. Zhu L. Li J. Maguire P. Zhou M. Sun H. (2018). Exploring the influence of ethical leadership on voice behavior: how leader-member exchange, psychological safety and psychological empowerment influence employees’ willingness to speak out. *Front. Psychol.* 9:1718 10.3389/fpsyg.2018.01718 PMC6143838 30258392

[B38] Hunsaker W. D. (2016). Spiritual leadership and organizational citizenship behavior: relationship with Confucian values. *J. Manag.* 13 206–225. 10.1080/14766086.2016.1159974

[B39] Javanmard H. (2012). The impact of spirituality on work performance. *Indian J. Sci. Technol.* 5 1961–1966. 10.17485/ijst/2012/v5i1/30966

[B40] Keyes M. W. Hanley-Maxwell C. Capper C. A. (1999). “Spirituality? It’s the core of my leadership”: empowering leadership in an inclusive elementary school. *Educ. Admin. Quart.* 35 203–237. 10.1177/00131619921968527

[B41] Kwon B. Farndale E. Park J. G. (2016). Employee voice and work engagement: macro, meso, and micro-level drivers of convergence? *Hum. Resour. Manage. R.* 26 327–337. 10.1016/j.hrmr.2016.04.005

[B42] Lavelle J. Gunnigle P. McDonnell A. (2010). Patterning employee voice in multinational companies. *Hum. Relat.* 63 395–418. 10.1177/0018726709348935

[B43] Lent R. W. Brown S. D. Hackett G. (1994). Toward a unifying social cognitive theory of career academic interest, choice, and performance. *J. Vocat. Behav.* 45 79–122. 10.1006/jvbe.1994.1027

[B44] Lepine J. A. Van Dyne L. (1998). Predicting voice behavior in work groups. *J. Appl. Psychol.* 83 853–868. 10.1037/0021-9010.83.6.853 30194864

[B45] Li J. Wu L. Z. Liu D. Kwan H. K. Liu J. (2014). Insiders maintain voice: a psychological safety model of organizational politics. *Asia Pac. J. Manag.* 31 853–874. 10.1007/s10490-013-9371-7

[B46] Liang J. (2014). Ethical leadership and employee voice: examining a moderated-mediation model. *Acta Psychol. Sin.* 46 252–264. 10.3724/SP.J.1041.2014.00252

[B47] Liang J. Farh C. I. Farh J. L. (2012). Psychological antecedents of promotive and prohibitive voice: a two-wave examination. *Acad. Manag. J.* 55 71–92. 10.5465/amj.2010.0176

[B48] Lin C. P. Tsai Y. H. Joe S. W. Chiu C. K. (2012). Modeling the relationship among perceived corporate citizenship, firms’ attractiveness, and career success expectation. *J. Bus. Ethics* 105 83–93. 10.1007/s10551-011-0949-z

[B49] Liu W. Zhu R. Yang Y. (2010). I warn you because I like you: voice behavior, employee identifications, and transformational leadership. *Leadersh. Quart.* 21 189–202. 10.1016/j.leaqua.2009.10.014

[B50] Madrid H. P. Patterson M. G. Leiva P. I. (2015). Negative core affect and employee silence: how differences in activation, cognitive rumination, and problem-solving demands matter. *J. Appl. Psychol.* 100 1887–1898. 10.1037/a0039380 26011721

[B51] Martinko M. J. Mackey J. D. Moss S. E. Harvey P. McAllister C. P. Brees J. R. (2018). An exploration of the role of subordinate affect in leader evaluations. *J. Appl. Psychol.* 103 738–752. 10.1037/apl0000302 29578738

[B52] Meng Y. Yang X. Qiu Y. (2018). From “external constraints” to “intrinsic motivation”: research on mechanism of spiritual leadership influences employees’ voice behaviors. *Hum. Resour. Dev.* 35 6–17. 10.16471/j.cnki.11-2822/c.2018.03.000

[B53] Morrison E. W. (2011). Employee voice behavior: integration and directions for future research. *Acad. Manag. Ann.* 5 373–412. 10.1080/19416520.2011.574506

[B54] Morrison E. W. (2014). Employee voice and silence. *Annu. Rev. Organ. Psychol.* 1 173–197. 10.1146/annurev-orgpsych-031413-091328

[B55] Mowbray P. K. Wilkinson A. Tse H. H. M. (2015). An integrative review of employee voice: identifying a common conceptualization and research agenda. *Int. J. Manag. Rev.* 17 382–400. 10.1111/ijmr.12045

[B56] Mucci N. Giorgi G. Roncaioli M. Perez J. F. Arcangeli G. (2016). The correlation between stress and economic crisis: a systematic review. *Neuropsychiatr. Dis. Treat.* 12 983–993. 10.2147/NDT.S98525 27143898 PMC4844458

[B57] Nurmi J. E. Aunola K. Salmela-Aro K. Lindroos M. (2003). The role of success expectation and task-avoidance in academic performance and satisfaction: three studies on antecedents, consequences and correlates. *Contemp. Educ. Psychol.* 28 59–90. 10.1016/S0361-476X(02)00014-0

[B58] Park J. Lee K. Lim J. I. Sohn Y. W. (2018). Leading with callings: effects of leader’s calling on followers’ team commitment, voice behavior, and job performance. *Front. Psychol.* 9:1706. 10.3389/fpsyg.2018.01706 30258386 PMC6143684

[B59] Pearce C. L. Sims H. P. Jr. (2002). Vertical versus shared leadership as predictors of the effectiveness of change management teams: an examination of aversive, directive, transactional, transformational, and empowering leader behaviors. *Group Dyn. Theory Res. Pract.* 6 172–197. 10.1037/1089-2699.6.2.172

[B60] Phuong N. V. Khoa T. T. Khanh H. D. Ho P. D. (2018). The role of leader’s spiritual leadership on organisation outcomes. *Asian Acad. Manag. J.* 23 45–68

[B61] Podsakoff P. M. MacKenzie S. B. Podsakoff N. P. (2012). Sources of method bias in social science research and recommendations on how to control it. *Annu. Rev. Psychol.* 63 539–569. 10.1146/annurev-psych-120710-100452 21838546

[B62] Premeaux S. F. Bedeian A. G. (2003). Breaking the silence: the moderating effects of self-monitoring in predicting speaking up in the workplace. *J. Manag. Stud.* 40 1537–1562. 10.1111/1467-6486.00390

[B63] Qian J. Song B. Jin Z. Wang B. Chen H. (2018). Linking empowering leadership to task performance, taking charge, and voice: the mediating role of feedback-seeking. *Front. Psychol.* 9:2025. 10.3389/fpsyg.2018.02025 30410461 PMC6209672

[B64] Qiu Y. Meng Y. Yang X. (2019). How does spiritual leadership inspire innovation?-the study on the Chain mediating effect of leader-member exchange and organizational identification. *East China Econ. Manag.* 33 44–50. 10.19629/j.cnki.34-1014/f.180209007

[B65] Reave L. (2005). Spiritual values and practices related to leadership effectiveness. *Leadersh. Quart.* 16 655–687. 10.1016/j.leaqua.2005.07.003

[B66] Rousseau D. M. (2001). Schema, promise and mutuality: the building blocks of the psychological contract. *J. Occup. Organ. Psychol.* 74 511–541. 10.1016/j.pubrev.2017.04.008

[B67] Ruck K. Welch M. Menara B. (2017). Employee voice: an antecedent to organisational engagement? *Publ. Relat. Rev.* 43 904–914. 10.1016/j.pubrev.2017.04.008

[B68] Russell R. F. (2001). The role of values in servant leadership. *Leadersh. Organ. Dev. J.* 22 76–84. 10.1108/01437730110382631

[B69] Seibert S. E. Kraimer M. L. Liden R. C. (2001). A social capital theory of career success. *Acad. Manag. J.* 44 219–237. 10.2307/3069452

[B70] Song X. Wu W. Hao S. Lu X. Zhang Y. Liu Y. (2017). On-work or off-work relationship? an engagement model of how and when leader-member exchange and leader-member guanxi promote voice behavior. *Chin. Manag. Stud.* 11 441–462. 10.1108/CMS-03-2017-0058

[B71] Stephens G. K. Szajna B. Broome K. M. (1998). The career success expectations scale: an exploratory and confirmatory factor analysis. *Educ. Psychol. Meas.* 58 129–141. 10.1177/0013164498058001011

[B72] Van Dyne L. Kamdar D. Joireman J. (2008). In-role perceptions buffer the negative impact of low LMX on helping and enhance the positive impact of high LMX on voice. *J. Appl. Psychol.* 93 1195–1207. 10.1037/0021-9010.93.6.1195 19025242

[B73] Vos A. D. Stobbeleir K. D. Meganck A. (2009). The relationship between career-related antecedents and graduates’ anticipatory psychological contracts. *J. Bus. Psychol.* 24 289–298. 10.1007/s10869-009-9107-3

[B74] Walumbwa F. O. Schaubroeck J. (2009). Leader personality traits and employee voice behavior: mediating roles of ethical leadership and work group psychological safety. *J. Appl. Psychol.* 94 1275–1286. 10.1037/a0015848 19702370

[B75] Wang H. Wu W. Liu Y. Hao S. Wu S. (2019). In what ways do Chinese employees speak up? An exchange approach to supervisor-subordinate guanxi and voice behavior. *Int. J. Hum. Resour. Manag.* 30 479–501. 10.1080/09585192.2016.1253030

[B76] Yan A. Xiao Y. (2016). Servant leadership and employee voice behavior: a cross-level investigation in China. *SpringerPlus* 5:1595. 10.1186/s40064-016-3264-4 27652168 PMC5026985

[B77] Yan P. (2018). Supervisor−subordinate guanxi and employee voice behavior: trust in supervisor as a mediator. *Soc. Behav. Personal.* 46 1169–1178. 10.2224/sbp.7098

[B78] Yu Y. (2008). China’s economic growth, global economic crisis and china’s policy responses. *Pak. Dev. Rev.* 47 337–355. 10.30541/v47i4Ipp.337-355

[B79] Zhang Y. Huai M. Xie Y. (2015). Paternalistic leadership and employee voice in china: a dual process model. *Leadersh. Quart.* 26 25–36. 10.1016/j.leaqua.2014.01.002

[B80] Zhu Y. Akhtar S. (2017). Leader trait learning goal orientation and employee voice behavior: the mediating role of managerial openness and the moderating role of felt obligation. *Int. J. Hum. Resour. Man.* 30 2876–2900. 10.1080/09585192.2017.1335338

